# Global Health Workforce Labor Market Projections for 2030

**DOI:** 10.1186/s12960-017-0187-2

**Published:** 2017-02-03

**Authors:** Jenny X. Liu, Yevgeniy Goryakin, Akiko Maeda, Tim Bruckner, Richard Scheffler

**Affiliations:** 1 Institute for Health and Aging, Department of Social and Behavioral Sciences, University of California, San Francisco, 3333 California Street, Suite 340, San Francisco, CA 94118 United States of America; 2 Health Division, Labour and Social Affairs, Organization for Economic Co-operation and Development, 2 rue Andre Pascal, 75775 Paris, Cedex 16 France; 3 School of Public Health, University of California, 635 E. Peltason Dr., Irvine, CA 92697-3957 United States of America; 4 School of Public Health, Goldman School of Public Policy, University of California, Berkeley, 50 University Hall MC7360, Berkeley, CA 94704 United States of America

**Keywords:** Health workforce, Labor market projections, Global health, J210, J230, J440

## Abstract

**Background:**

In low- and middle-income countries, scaling essential health interventions to achieve health development targets is constrained by the lack of skilled health professionals to deliver services.

**Methods:**

We take a labor market approach to project future health workforce demand based on an economic model based on projected economic growth, demographics, and health coverage, and using health workforce data (1990–2013) for 165 countries from the WHO Global Health Observatory. The demand projections are compared with the projected growth in health worker supply and the health worker “needs” as estimated by WHO to achieve essential health coverage.

**Results:**

The model predicts that, by 2030, global demand for health workers will rise to 80 million workers, double the current (2013) stock of health workers, while the supply of health workers is expected to reach 65 million over the same period, resulting in a worldwide net shortage of 15 million health workers. Growth in the demand for health workers will be highest among upper middle-income countries, driven by economic and population growth and aging. This results in the largest predicted shortages which may fuel global competition for skilled health workers. Middle-income countries will face workforce shortages because their demand will exceed supply. By contrast, low-income countries will face low growth in both demand and supply, which are estimated to be far below what will be needed to achieve adequate coverage of essential health services.

**Conclusions:**

In many low-income countries, demand may stay below projected supply, leading to the paradoxical phenomenon of unemployed (“surplus”) health workers in those countries facing acute “needs-based” shortages. Opportunities exist to bend the trajectory of the number and types of health workers that are available to meet public health goals and the growing demand for health workers.

**Electronic supplementary material:**

The online version of this article (doi:10.1186/s12960-017-0187-2) contains supplementary material, which is available to authorized users.

## Background

The Sustainable Development Goals (SDGs) for health and well-being lay out ambitious targets for disease reduction and health equity for 2030, including universal health coverage (UHC) [1]. Health systems are highly labor intensive, and health workers play a key role in performing or mediating most of the health system functions. Thus, an effective health care delivery system depends on having both the right number and the appropriate mix of health workers, and on ensuring that they have the required means and motivation to perform their assigned functions well [2].

In many low- and middle-income countries, efforts to scale-up health services to achieve UHC and health development goals are confronted by acute shortages and inequitable distribution of skilled health workers that present a binding constraint to delivering essential health services [3, 4]. These countries face a “crisis in human resources for health” that can be described in terms of (1) availability, which relates to the supply of qualified health workers; (2) distribution, which relates to the recruitment and retention of health workers where they are needed most; and (3) performance, which relates to health worker productivity and the quality of the care they provide [5]. Multiple conditions contribute to this problem, including inadequate education and training capacity, negative work environments, weak human resources regulatory and management systems, and inadequate financial and non-financial incentives [6, 7]. National policymakers, researchers, and international agencies have called attention to this global shortage and maldistribution of the health workforce, and for governments to make concerted efforts to address these challenges in order to achieve UHC [4, 8].

Given the criticality of the health workforce in the health system, and substantial time and resources invested to educate and develop skilled health workers, it is crucial to understand the factors that affect the size of the future health workforce and plan appropriately today. Traditional approaches to addressing human resource constraints in the health sector have focused on “needs-based” workforce planning, which estimates health workforce requirements based on a country’s disease burden profile and commensurate scale-up of education and training capacities to increase the supply of health workers to provide those services [9, 10]. In this approach, health workforce density has been found to be associated with decreases in maternal and infant mortality rates [2, 11], as well as in the total burden of disease as measured in disability-adjusted life years (DALYs) [12]. Using this approach, the World Health Organization (WHO) estimates that a health workforce density of around 4.45 health workers per 1000 population corresponds to the median level of health workforce density among countries that have achieved, or have come close to achieving, UHC [13]. Policy makers could then identify the production capacity and associated financing necessary to increase the stock of health workers to meet these health service requirements [4, 13].

However, this needs-based approach neglects other important factors that influence the size of the health workforce, notably labor market dynamics that are defined by demand and supply interactions [5, 14]. It should not be assumed that labor markets always “clear,” in other words that the supply and demand for workers perfectly match. There are a number of reasons for an imbalance between the demand and supply for workers. For example, prices may not adjust easily due to fixed wage rates established by legislative or bureaucratic processes, or may be tied to civil service schedules that make them relatively insensitive to the numbers of health workers employers seek to hire or who are willing to be employed. Other institutional rigidities, such as regulatory guidelines and trade unions, can also restrict the extent to which the number of workers demanded or supplied responds to price signals. These situations can lead to either a shortage (i.e., quantity demanded exceeds the quantity supplied) or surplus (i.e., quantity demanded falls behind the quantity supplied) of health workers. Further, the number of health workers estimated to be “needed” to achieve the national health goal of UHC may not necessarily coincide with the demand for health workers due to economic capacity and other market conditions in the health system. Countries may also face unemployment among health workers when the supply of health workers exceeds demand generated by the country’s underlying economic capacity to employ them. A labor market analysis will help to identify such mismatch of labor supply and demand, and lead to more effective policy design to address these issues [15].

This study estimates the demand for health workers in 2030 (the target achievement year of the SDGs) as a function of economic, demographic, and health coverage factors based on an economic model. The model assumes no change in technology or organization of health services and thus projects the demand for health care as if the current system of healthcare and technology remains the same in 2030. We then compare this demand projection with the supply and the “needs” projections (based on the WHO SDG threshold density of 4.45 health workers per 1000 population [13]), and discuss the potential policy implications of the findings.

## Methods

### Theoretical framework

The demand for health workers reflects the willingness to pay of the purchasers of healthcare (e.g., government, private sector firms), which in turn drives the demand for employing health workers in clinics, hospitals, public health centers, and other parts of the health system. The demand for health workers is influenced by factors including household income (i.e., the ability of consumers to purchase health services), the fiscal capacity of the government to support the health system and employ public sector workers, demographic and epidemiologic conditions of the population (e.g., aging and burden of disease that determine the relative types of health services consumers want), and the level of health coverage in terms of risk pooling and financial protection available to enable consumers to access and utilize healthcare at times of need.

The supply of health workers can be defined as the total number of health professionals with the appropriate skills and qualifications who are willing to enter into job market in the health sector and find acceptable jobs. Labor economics predicts that, as the level of compensation offered increases, more qualified workers should be willing to become employed as a health professional [16, 17]. In turn, higher wages encourages more students to apply for health professional education, and increases the demand for medical training and eventually the number of skilled professionals available. In a global labor market where workers’ skills may be transferable across country boundaries, migration flows also play an important role in determining the supply of health workers within a country. In particular, outflows of health professionals from low- and middle-income countries to other, more attractive markets offering better compensation have been identified as one of the biggest challenges facing health systems [6, 18].

**Fig. 1:**
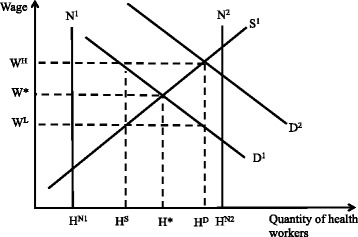
Health worker static labor market theoretical framework. Legend: Demand (D) and supply (S) interact to determine the number of workers (H*) that will be employed at a market wage rate (W*). At a wage rate (W^L^) that is lower than the market optimum (W*), a shortage of workers results, and the number of workers demanded (H^D^) exceeds the number supplied (H^S^). To alleviate shortages in this market, either (1) additional compensation could be given to increase wages to W* and attract more workers into the market, or (2) the production of workers could be increased such that supply shifts outward (S^2^) and the quantity demand (H^D^) is achieved while keeping wages at W^L^

Traditional labor economic analyses assume that, in well-functioning labor markets, disequilibrium (i.e., imbalances between demand and supply) is short-lived. A core assumption is that the wage rate is flexible and freely adjusts the incentives to both employers and health workers, influencing their employment behaviors and preferences such that equilibrium is restored. Figure 1 depicts a static health worker labor market in which employers’ demand (D) to employ health workers interacts with the health workers available to supply (S^1^) their labor to determine the market wage rate (W*) and the number of workers (H*) that will be employed at that rate. Countries face a binding constraint on the amount of financing available to employ more health workers, and resource constraints are more severe in lower-income countries. A shortage of workers results, for example, when a wage rate (W^L^) is lower than the market optimum (W*), or when the number of workers supplied (H^S^) falls below the number demanded (H^D^). All else being equal, shortages in this market could be alleviated through (1) additional compensation to increase wages to W* and attract more workers into the market; and/or (2) increasing the production of workers or import of workers from external markets to shift the supply curve outward (S^2^) while keeping wages at W^L^.

In reality, markets can fail to “clear” because prices are either not flexible, or demand and/or supply does not readily adjust to price signals. Both types of rigidities are common in the health labor market [14]. First, price of labor in the health sector is often not flexible because wages in the public health sector (often the largest employer in many countries) are usually set by legislative processes and tied to civil servant pay scales. Second, health professional associations (especially for physicians) use their bargaining power to restrict labor supply and negotiate set wage rates. Third, the regulation of health services, such as licensing by professional bodies and governmental jurisdictions, to ensure quality standards and monitoring results in additional rigidities in the ability of workers to become employed wherever positions may be available.

### Projecting demand

The demand model builds on a previous economic model for projecting physician numbers [19]. We apply a similar theoretical approach but incorporate factors in addition to economic growth that are expected to influence demand for health workers, including population demographic structure and health coverage. We also use more recent and robust health workforce data, which are the result of concerted efforts by the WHO to gather cross-national data on workforce numbers since 1990. Because the demand model requires rich historical data on health worker densities, separate models for nurses/midwives and all other health professionals could not be estimated; data for these cadres were insufficient to produce demand projections. Rather, we first predicted the number of physicians from the demand model, and then applied constant ratios of physicians to other cadres to obtain estimates of nurses/midwives and all other health workers (AOWs). The total projected number of health workers therefore reflects the sum of the estimates for physicians, nurses/midwives, and AOWs.

In most health systems, spending on health workforce wages and benefits represents a significant share of total health expenditures [20]. Previous studies indicate that overall economic growth, as measured by national income, is the best predictor of health expenditures from which the demand for health workers is derived [21, 22]. In other words, spending on healthcare tends to increase as overall income increases, which in turn suggests that more workers can be employed to deliver health services [19, 21].

To our knowledge, few have previously projected future health workforce labor market demand. Owing to data requirements, early efforts largely focused on specific developed countries for which data on health workers are more readily available [23]. Leveraging efforts to obtain cross-national and longitudinal data on health workers, Scheffler et al. [19] were the first to forecast the demand, need, and supply of physicians for 158 countries with suitable data. While notable in the scope of global coverage, their resulting model relied on only one model parameter input—gross national income—to generate projections.

Our demand model expands on previous methods developed by Scheffler et al. [19]. In addition to income (i.e., gross domestic product (GDP) per capita), we included measures for demographic and health coverage patterns that also drive demand for health workers [21, 22, 24]. The size of the population aged 65 or over was used as an indicator of aging, which is known to increase the demand for health services [24]. We also included private per capita household out-of-pocket (OOP) spending on medical care as a proxy indicator for the extent of social protection for healthcare spending. While overall healthcare spending may trend upward with national income, the portion spent OOP is largely determined by the level of health coverage by health insurance, government subsidies, and other forms of risk pooling and financial protection [25]. Less generous health coverage leaves individuals to pay more OOP, which is expected to lower the demand for and use of health services. Thus, we expect higher OOP health spending to be correlated with lower demand for health workers. We exclude additional structural factors affecting the labor market, such as attrition, training capacity, labor regulations, and migration, as these data are largely unavailable across countries or over time.

The following section provides a summary description of the estimation model. A detailed and technical description of the demand projection methodology, model specification choice, and imputation for missing data is provided in Additional file 1: Annex A. Due to the missing data problems for nurses/midwives and AOWs, the economic model was first used to predict the demand for physicians. Systematic ratios were then applied to predicted physician densities to estimate the number of nurses/midwives and AOWs.

The economic model specifies physician density (dependent variable) as a function of GDP per capita, OOP per capita, and the size of the population over 65 years. Given data availability constraints, we included a vector of dummy variables by country (i.e., fixed effects) to account for constant differences in characteristics across countries. All independent variables were lagged up to 5 years to ensure that historical levels of these measures predict future worker density numbers, and which allows time for such factors to work through the economy and affect the labor market. A stepwise approach was used to select the combination of year lags of the different predictors that maximized the predictive power of each variable. The resulting optimal model is as follows:

1 $$ \begin{array}{l} \ln \left( physicians\  per\  1000\ {population}_{i t}\right)={\beta}_0+{\beta}_1\ast \ln \left( GDP\  per\ {capita}_{i t- 1}\right)\\ {}+{\beta}_2\ast \ln \left( GDP\  per\ {capita}_{i t- 4}\right)+{\beta}_3\ast \ln \left( GDP\  per\ {capita}_{i t- 5}\right)+{\beta}_4\ast \ln \left( OOP\  per\ {capita}_{i t-2}\right)+{\beta}_5\ast \ln \left( Pop{65}_{i t- 3}\right)+{\mu}_i+{\xi}_{i t}\end{array} $$

where *μ*_*i*_ represents the vector of country dummy variables, *ξ*_*it*_ is the disturbance term, and *β* coefficients are unknown parameters to be estimated from the model. To allow for a more flexible functional form, quadratic terms for income and health spending indicators were investigated but ultimately excluded because they yielded little additional predictive value.

Equation 1 was fit through a generalized linear model (GLM). Estimated coefficients from the regression model were then applied to the future values of each predictor variable to compute the future predicted physician density.

Alternative model specifications were explored, which included different ways in which input parameters could be calculated (e.g., percentage of the population aged 65+, OOP health spending as a percentage of total health expenditures). To select the appropriate model, the data were split into an “initializing” dataset (data years 1995–2004) and an “attesting” dataset (data years 2005–2013) with which to assess the precision of predicted values resulting from different specifications [26]. The model in Eq. 1 yielded predictions with the lowest mean errors.

We conducted two additional sensitivity analyses of the projections of physician demand resulting from alternative input parameters for the optimal demand model chosen. First, we assessed the stability of the predictions to alternative estimated future values of GDP per capita (US$2010) obtained from the Economic Research Service International Macroeconomic Data Set published by the United States Department of Agriculture (USDA) [27]. There was a relatively small (9%) difference in the total estimated shortages in 2030 based on the two methods (15.6 million with the main method we used, and 17.0 million using USDA numbers).

Second, we examined the possible upper and lower bounds of the predictions resulting from high and low future estimates of the size of the population over 65 years generated by the United Nations Population Division [28]. Because population is the largest driver of demand in our model, we can obtain predicted total health worker deficits that may result from population growth among people older than 65 that is higher and lower than expected, compared to the median estimate that is presented in the main results. These alternative low and high estimates (shown in Additional file 1: Appendix Figure A1) indicate a tight band for the resulting predicted values.

### Projecting supply

The supply of health workers was projected to 2030 using historical data to predict the changes in health worker densities (per 1000 population) for each country. We assumed that the historical growth rate of health worker densities for each country would continue to 2030 at the same rate each year. This assumes that supply growth only trends with time, which may be plausible if the health labor market is relatively rigid, for example, due to the strong influence of professional associations and trade unions in the sector. Given that data were only available beginning from 1990 and that many countries only have a few data points, the growth rate method also enables us to obtain projections using minimal empirical data inputs.

We separately estimate densities for physicians and nurses/midwives for each country from time *t* = {1990, … 2013} using the following equations:

2 $$ Physicians\  per\  1000\ {population}_t={\alpha}_0 + {\alpha}_1*{year}_t + {\varepsilon}_t $$

3 $$ Nurses/ midwives\  per\  1000\ {population}_t={\beta}_0 + {\beta}_1*{year}_t + {\varepsilon}_t $$

where *ε*_*t*_ is the random disturbance term and *α*_*0*_, *β*_*0*_, *α*_*1*_, and *β*_*1*_ are unknown parameters; the last two parameters represent the linear growth rates estimated from the model.

For countries where more than two historical data points were available for physician and nurse/midwife densities, the future projections of worker densities were predicted based on the model parameter estimates. This occurred in 136 and 81 countries, respectively, out of the total 165 countries in our sample.

For countries with fewer than two historical data points or where the linear regression predictions yielded implausible values, several alternative approaches were used to estimate future supply (see Additional file 1: Annex A for details). Briefly, for 72 and 118 countries for physicians and nurses/midwives, respectively, the median density growth rate for the country’s region and income group was applied to the most recent data year to obtain future year predicted values. In a number of countries where no empirical data for nurses/midwives was available but information on physicians was available, a global ratio of 2.517 nurse/midwives to physicians was applied to obtain the estimate for nurse/midwife density. The constant ratio for AOWs was then similarly applied to obtain an estimate for all other health workers. Supply estimates for all three types of health workers were summed to obtain the total supply of health workers per country (see Additional file 1: Annex A for more details).

We explored alternatively specifying Eqs. 2 and 3 as log-linear models, which assumes an exponential functional form. The resulting projections from the exponential specification yielded estimates that were magnified compared to those from the linear specification, exaggerating both positive and negative trends. Coupled with the sparse number of data points for many lower-income countries, resulting predicted values appeared to be less stable. Within-sample specification tests were also not possible given the lack of sufficient time trend data for each country. We therefore adopted the more conservative linear specification.

### Data

Country-level data were collated from multiple sources. Historical data on physician and nurse/midwife densities (1990–2013) were obtained from the WHO Global Observatory Health database [29]. Historical and projected total population and population aged 65 and over were obtained from the United Nations Population Division [28].

We used the World Bank Development Indicator database to retrieve historical (1995–2013) GDP per capita (in constant 2011 dollars, purchasing power parity (PPP) values), total health expenditures per capita (in constant 2011 international dollars, PPP values), and the share of health spending OOP [30]. Projected real GDP per capita through 2030 were obtained from the analysis carried out by Patrick Huang-Vu Eozenou (see Additional file 1: Annex B for details). Historical data on OOP expenditures per capita (1995–2013) were calculated from health expenditure and OOP percentage data. Estimates for future OOP per capita were obtained by projecting the OOP percentage of total health spending from 2014 to 2030 and selecting the models giving the smallest predicted error for each country (see Additional file 1: Annex A for details). Projections of future workforce demand were made for 165 countries for which both demand and supply input data were available.

To obtain the estimates for nurses and midwives and all other workers, we use the approach taken by WHO to fill missing data by multiplying the projected physician density for each country by a constant 2.517 ratio of nurses and midwives to physicians based on the WHO Global Observatory data [29]. This estimation assumes that the skills mix for healthcare workers will stay constant and is the same for all countries. To obtain estimates of AOWs, we similarly applied constant multipliers, but did so stratified according to World Bank income groups (high income = 0.373; upper middle income = 0.406; lower middle income = 0.549; low income = 0.595). These ratios were derived by WHO from the ratios of nurses/midwives to physicians in 2013 (the most recent data year) and multipliers used for AOWs [31].

## Results

Table 1 summarizes the estimated coefficients from the demand model. The income elasticity for physicians per capita is 0.23, which indicates that a 10% increase in per capita GDP (lagged 1 year) is correlated with a 2.3% increase in physician density. The elasticity of physician density with respect to OOP health expenditures is negative (consistent with our hypothesis) and significant, indicating that a 10% increase in OOP expenditures (lagged 2 years) is associated with a 1.0% decrease in physician density. In addition, a 10% increase in the size of the population aged 65 or older (lagged 3 years) increases physician density by 5.2%.

**Table 1:** Demand model GLM estimates

	Ln(physicians per 1000)	95% confidence interval
Ln(*GDP per capita*_*t−1*_)	0.231***	0.076, 0.387
	(0.079)	
Ln(*GDP per capita*_*t−4*_)	0.531***	0.253, 0.810
	(0.142)	
Ln(*GDP per capita*_*t−5*_)	−0.518***	−0.780. −0.256
	(0.133)	
Ln(*OOP per capita*_*t−2*_)	−0.099***	−0.144, −0.054
	(0.023)	
Ln(*Pop65*_*t−3*_)	0.516***	0.372, 0.660
	(0.073)	
Constant	−9.882***	−11.47, −8.29
	(0.810)	
Observations	1179	
Country fixed effects	YES	
Log-likelihood	658.1	

*** *p*<0.01

Table 2 presents projected health worker demand, supply, and net differences (supply-demand) by region and income group for 2013 and 2030. The net differences between the number demanded and the number supplied is then shown as either shortage (negative) or surplus (positive). These data projections for each country are further stratified according to resulting overall shortage or surplus of health workers (again defined as the difference in supply and demand) and are shown in Tables 3 and 4, respectively, for 2013 and 2030.

**Table 2:** Estimated demand and supply of health workers, by regions and income group, 2013 and 2030

	2013 (165 countries)			2030 (165 countries)			# Countries in each group
	Demand	Supply	Diff (S-D)	Demand	Supply	Diff (S-D)	
WHO Region							
Africa	1,106,183	1,874,830	768,647	2,404,807	3,066,666	661,859	43
Americas	8,826,933	8,385,480	−441,453	15,288,610	12,742,856	−2,545,753	28
Eastern Mediterranean	3,057,524	2,690,443	−367,080	6,201,515	4,611,408	−1,590,107	15
Europe	14,178,009	12,692,401	−1,485,607	18,158,772	16,803,264	−1,355,508	50
South-East Asia	5,964,318	5,772,250	−192,067	12,206,786	10,168,591	−2,038,195	8
Western Pacific	15,133,290	10,294,627	−4,838,663	25,894,849	17,261,342	−8,633,507	21
WB Region							
East Asia and Pacific	15,481,985	11,141,638	−4,340,347	26,546,027	18,250,702	−8,295,325	23
Europe and Central Asia	14,007,183	12,594,176	−1,413,007	17,844,850	16,640,618	−1,204,233	48
Latin America	4,526,235	4,140,233	−386,002	8,374,987	5,784,767	−2,590,219	26
Middle East & North Africa	2,517,001	2,570,885	53,884	4,913,419	3,846,948	−1,066,471	15
North America	4,300,699	4,245,248	−55,451	6,913,623	6,958,089	44,466	2
South Asia	6,494,350	5,357,579	−1,136,771	13,459,980	10,293,688	−3,166,292	8
Sub-Saharan Africa	938,804	1,660,273	721,470	2,102,453	2,879,315	776,862	43
WB Income group							
Low	1,400,074	692,757	−707,317	1,400,074	1,384,576	−15,498	29
Lower-middle	21,682,581	9,867,919	−11,814,662	21,682,581	17,958,943	−3,723,638	44
Upper-middle	33,291,730	13,764,139	−19,527,591	33,291,730	21,362,033	−11,929,697	46
High	23,780,953	17,385,217	−6,395,736	23,780,953	23,948,576	167,622	46
World	48,266,256	41,710,032	−6,556,224	80,155,338	64,654,127	−15,501,211	165

Health worker refers to physicians, nurses/midwives, and all other health workers. Demand for nurses/midwives was calculated assuming a ratio of 2.517 nurses/midwives to one physician. Demand and supply of all other health workers was calculated assuming a ratio of 3.517 doctors and nurse/midwives times a World Bank income group-specific multiplier. Supply of physicians and nurses/midwives was projected based on the country-specific linear growth rates of physicians and nurses per 1000 population

**Table 3:** Estimated health worker shortages and surpluses by regions and income group, 2013

	Shortage countries					Surplus countries				
	Demand	Supply	Need	Difference (S–D)	# Countries	Demand	Supply	Need	Difference (S–D)	# Countries
WHO Region										
Africa	32,357	20,570	168,664	−11,787	3	1,073,826	1,854,259	5,722,406	780,434	40
Americas	6,612,863	5,111,374	3,872,984	−1,501,490	18	2,214,070	3,274,107	1,566,639	1,060,037	10
Eastern Mediterranean	2,325,668	1,511,359	2,576,703	−814,309	9	731,856	1,179,084	1,221,066	447,229	6
Europe	9,432,898	6,916,358	3,504,234	−2,516,540	29	4,745,111	5,776,043	2,124,299	1,030,933	21
South-East Asia	5,542,630	4,847,018	10,129,877	−695,613	3	421,687	925,233	2,303,207	503,546	5
Western Pacific	13,132,605	7,142,858	10,183,203	−5,989,747	6	2,000,685	3,151,769	1,355,351	1,151,084	15
WB Region										
East Asia and Pacific	13,132,605	7,142,858	10,183,203	−5,989,747	6	2,349,380	3,998,780	3,509,697	1,649,400	17
Europe and Central Asia	9,262,072	6,818,133	3,453,923	−2,443,939	27	4,745,111	5,776,043	2,124,299	1,030,933	21
Latin America	2,662,078	1,414,236	1,935,612	−1,247,842	17	1,864,157	2,725,997	1,351,392	861,841	9
Middle East & North Africa	1,617,766	1,177,244	1,160,165	−440,522	9	899,235	1,393,641	1,194,530	494,406	6
North America	3,950,785	3,697,138	1,937,372	−253,647	1	349,914	548,110	215,247	198,196	1
South Asia	6,421,358	5,279,358	11,596,725	−1,142,000	5	72,992	78,221	148,861	5230	3
Sub-Saharan Africa	32,357	20,570	168,664	−11,787	3	906,447	1,639,703	5,748,942	733,256	40
WB Income group										
Low	395,927	223,855	1,699,364	−172,072	5	241,657	468,902	3,162,540	227,245	24
Lower middle	8,834,452	6,850,983	12,800,744	−1,983,469	17	2,063,083	3,016,936	4,804,549	953,853	27
Upper middle	15,462,673	8,497,379	10,939,972	−6,965,294	20	3,577,879	5,266,760	3,677,217	1,688,881	26
High	12,385,969	9,977,320	4,995,585	−2,408,649	26	5,304,614	7,407,897	2,648,661	2,103,283	20
World	37,079,021	25,549,537	30,435,665	−11,529,485	68	11,187,234	16,160,495	14,292,968	4,973,261	97

**Table 4:** Estimated health worker shortages and surpluses by regions and income group, 2030

	Shortage countries					Surplus countries				
	Demand	Supply	Need	Shortage	# Countries	Demand	Supply	Need	Surplus	# Countries
WHO Region										
Africa	643,548	453,757	2,423,284	189,791	13	1,761,259	2,612,909	6,487,188	851,649	30
Americas	5,294,324	2,177,226	2,270,537	3,117,097	17	9,994,286	10,565,630	3,975,926	571,344	11
Eastern Mediterranean	5,735,711	3,976,303	4,248,811	1,759,408	12	465,804	635,105	806,814	169,301	3
Europe	8,813,848	6,485,872	2,740,579	2,327,976	32	9,344,924	10,317,392	3,045,689	972,468	18
South-East Asia	11,420,891	9,039,083	12,091,674	2,381,808	3	785,895	1,129,508	2,621,313	343,613	5
Western Pacific	23,359,616	13,836,069	11,088,037	9,523,547	10	2,535,233	3,425,273	1,182,439	890,040	11
WB Region										
East Asia and Pacific	23,359,616	13,836,069	11,088,037	9,523,547	10	3,186,411	4,414,633	3,646,462	1,228,222	13
Europe and Central Asia	8,499,926	6,323,225	2,676,878	2,176,701	30	9,344,924	10,317,392	3,045,689	972,468	18
Latin America	5,294,324	2,177,226	2,270,537	3,117,097	17	3,080,663	3,607,541	1,555,339	526,878	9
Middle East & North Africa	4,574,811	3,342,787	2,615,168	1,232,024	13	338,608	504,161	417,742	165,553	2
North America	0	0	0	0	0	6,913,623	6,958,089	2,420,587	44,466	2
South Asia	13,325,263	10,153,540	14,091,101	3,171,723	5	134,717	140,148	157,290	5431	3
Sub-Saharan Africa	213,998	135,463	2,121,201	78,535	12	1,888,455	2,743,852	6,876,260	855,397	31
WB Income group										
Low	1,142,167	787,953	4,149,362	354,214	14	257,907	596,623	2,899,686	338,716	15
Lower middle	19,143,815	14,797,698	16,063,131	4,346,117	23	2,538,766	3,161,245	5,877,124	622,479	21
Upper middle	28,403,598	15,305,990	12,465,315	13,097,608	23	4,888,132	6,056,042	3,469,462	1,167,910	23
High	6,578,358	5,076,669	2,185,113	1,501,689	27	17,202,596	18,871,907	5,873,098	1,669,311	19
World	55,267,937	35,968,311	34,862,922	−19,299,627	87	24,887,401	28,685,817	18,119,370	3,798,416	78

Table 2 shows that by 2030, worldwide demand for health workers will increase to 80 million, but only 65 million will be supplied, amounting to a net shortage of some 15 million workers. This is over a twofold increase over the estimated shortage of nearly 7 million workers in 2013. In 2030, the largest net shortages are predicted to occur in the East Asia and Pacific (8.3 million), followed by South Asia (3.2 million), Latin America and Caribbean (2.6 million), and Europe and Central Asia (1.2 million). In terms of income groups, the highest level of shortages in 2030 will occur in upper middle-income countries, followed by lower-middle-income countries. These countries are likely to experience relatively higher rates of economic growth and population aging, which will generate substantial demand for health workers that may not be adequately met by the increase in supply of health workers. In contrast, countries in sub-Saharan Africa region show a surplus of health worker (0.8 million), indicating that these countries may experience unemployment or under-employment of health workers. Paradoxically, as will be discussed below, this labor surplus in sub-Saharan Africa will occur in the context where the demand and supply of health workers remain significantly below the WHO SDG threshold of 4.45 workers per 1000 population needed to achieve UHC.

Table 3 shows that in 2013, 68 countries showed an aggregate shortage of 11.6 million health workers, while 97 countries showed a surplus of 5.0 million. Table 4 shows that by 2030, some 87 countries will experience health worker shortages of 19.3 million, while the surplus of health workers will reduce to 3.8 million workers across 78 countries. The substantial portion of countries exhibiting a surplus of health workers in 2030 suggest that such excesses of workers beyond what employers will demand may fuel global migration of health workforce, especially if the surplus workers have skills profiles that meet the demand from the countries facing health workforce shortage. Otherwise, these surplus workers will add to unemployment in their respective countries.

Table 5 compares the supply projections of health workers with the needs-based projections of the number of health workers required to meet basic health service utilization targets as defined by WHO for the same 165 countries for which we have demand estimates [31]. The shortage (negative) or surplus (positive) of health workers according to the need criteria is similarly calculated as the net difference between the number needed and the number supplied for each region or income group. By 2030, based on the need-based model, the largest health worker shortages will occur in low- and lower middle-income countries, particularly in the sub-Saharan Africa and South Asia regions. Low-income countries face a situation in which both the demand and supply curves for predicted health worker densities fall below the WHO need threshold. In these countries, increasing the supply of health workers to the WHO threshold level will not be sustainable via the market alone, since the demand generated in the labor market will not be sufficient to employ higher numbers of health workers.

**Table 5:** Estimated health workforce supply versus “need” by regions and income group, 2013 and 2013

	2013 (165 countries)			2030 (165 countries)			# Countries in Category
	Supply (S)	Need (N)	Diff (S–N)	Supply (S)	Need (N)	Diff (S–N)	
WHO Region							
Africa	1,874,830	5,891,071	−4,016,241	3,066,666	8,910,473	−5,843,806	43
Americas	8,385,480	5,439,623	2,945,857	12,742,856	6,246,463	6,496,393	28
Eastern Mediterranean	2,690,443	3,797,769	−1,107,326	4,611,408	5,055,625	−444,217	15
Europe	12,692,401	5,628,533	7,063,868	16,803,264	5,786,268	11,016,996	50
South-East Asia	5,772,250	12,433,083	−6,660,833	10,168,591	14,712,987	−4,544,397	8
Western Pacific	10,294,627	11,538,553	−1,243,926	17,261,342	12,270,476	4,990,867	21
WB Region							
East Asia and Pacific	11,141,638	13,692,899	−2,551,261	18,250,702	14,734,499	3,516,203	23
Europe and Central Asia	12,594,176	5,578,223	7,015,953	16,640,618	5,722,567	10,918,050	48
Latin America	4,140,233	3,287,004	853,229	5,784,767	3,825,876	1,958,892	26
Middle East & North Africa	2,570,885	2,354,695	216,190	3,846,948	3,032,910	814,038	15
North America	4,245,248	2,152,619	2,092,629	6,958,089	2,420,587	4,537,501	2
South Asia	5,357,579	11,745,586	−6,388,007	10,293,688	14,248,390	−3,954,702	8
Sub-Saharan Africa	1,660,273	5,917,606	−4,257,333	2,879,315	8,997,462	−6,118,146	43
WB income groups							
Low	692,757	4,861,904	−4,169,147	1,384,576	7,049,048	−5,664,472	29
Lower middle	9,867,919	17,605,293	−7,737,374	17,958,943	21,940,256	−3,981,313	44
Upper middle	13,764,139	14,617,189	−853,050	21,362,033	15,934,777	5,427,256	46
High	17,385,217	7,644,247	9,740,970	23,948,576	8,058,211	15,890,364	46
World	41,710,032	44,728,633	−3,018,601	64,654,127	52,982,292	11,671,836	165

Health worker “need” refers to the WHO SDG threshold density of 4.45 health workers per 1000 population [31]

**Fig. 2:**
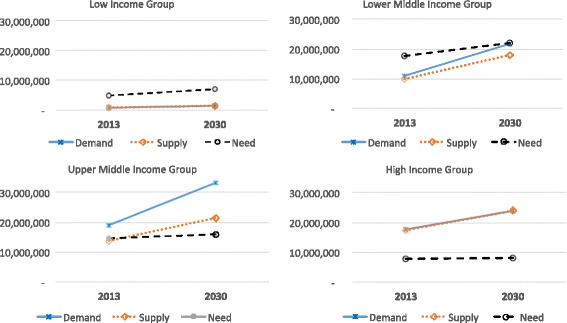
Trends in the projected demand, supply, and need-based number of health workers by World Bank income group, 2013–2030. Legend: Middle-income countries are predicted to experience the largest and most rapidly increasing demand and subsequent labor market shortages over this time period. The average annual growth in the supply of health workers is lower in high- and upper middle income countries than in the lower-middle income countries, but the comparatively higher growth in demand will lead to the largest health worker shortages in the labor market in upper middle-income countries. The growth in the supply for workers is predicted to be the slowest in low-income countries, but the growth in demand is also slow. As a result, the net shortage of health workers in low-income countries will reduce by 2030, but still fall significantly below the threshold level (4.45 health workers per 1000 population) estimated by WHO [31] to be required to meet the basic health care needs

In summary, trends in the predicted health worker demand, supply, and needs by income level are illustrated in Fig. 2. Growth in both demand for and supply of health workers is predicted to be the slowest in low-income countries, and these are projected to remain significantly below the WHO SDG threshold of 4.45 workers per 1000. As a result, these low-income countries might experience a paradoxical situation in which they face a shortage of health workers needed to provide basic health services, but also have unemployed health workers due to the limited national capacity to employ the available supply of workers. Middle-income countries are predicted to experience the largest increase in net shortages (per demand projections) over this time period, reaching 3.7 million workers in lower-middle-income countries and 11.9 million workers in upper middle-income countries by 2030. Although these countries will generate sufficient demand for health workers that meet and exceed the WHO SDG threshold density, their challenges will be in producing sufficient numbers of qualified health workers to meet projected demand. The model predicts that high-income countries would have a relatively balanced growth in both demand and supply of health workers. However, it should be pointed out that the supply projections used in this analysis used only the net increase in the supply of health workers and did not take into account changes in the attrition and retirement rates of the health workers. Estimates for the European Union that take into account these workforce dynamics suggest that many high-income countries will face substantial shortages of health workers by 2020 due to rapid aging of the current stock of health workers if the rate of production is not increased to compensate for anticipated higher exit rates [32].

## Discussion

Policymakers must allocate resources and set priorities today based on expectations of future need and capacity to support health workers. Our projections of health worker demand show that predicted trends in the labor market will likely enable many countries to employ more health workers, but that the supply of health workers will not keep pace for about half the countries in the world. By 2030, we estimate a net global demand-based shortage of over 15 million health workers. Labor shortages are predicted to be most severe in middle-income countries and for the East Asia and Pacific region, which is anticipated to have a large increase in demand due to relatively more robust economic growth, rapid population growth and aging, and modest social protection for OOP private health spending. The smallest demand-based shortages are predicted for low-income countries, and particularly for sub-Saharan Africa where neither the supply of nor the demand for workers is expected to grow substantially.

By contrast, most of the middle- and high-income countries are expected to produce enough health workers to meet or exceed the needs-based threshold of 4.45 workers per 1000. The number of health workers demanded in upper middle- and high-income countries already meets or exceeded the 4.45 health worker per 1000 population threshold in 2013 [31], and aggregate demand in lower middle-income countries is also projected to surpass this need-based threshold by 2030. However, these countries may face challenges in generating sufficient supply of health workers to meet this demand.

In many middle-income countries where we predict robust economic growth and aging, demand shortages may arise due to the country’s inability to scale the supply of health workers to meet the rapid growth in demand for healthcare. Since it takes considerable time to educate qualified health professionals, this labor shortage may lead to wage increases that attract workers from elsewhere, often from lower-income countries to higher-income countries, which could exacerbate health worker deficits in low-income sending countries. These challenges may be particularly pronounced in emerging economies where growth in demand will add considerable pressure to produce more health workers of acceptable quality or accept health workers from other countries.

Comparing needs- and demand-based projections underscores the importance of understanding whether shortages stem from supply or economic demand constraints, or both. In low-income countries where supply and demand are both low, there may be neither enough employment capacity nor adequate numbers of health workers to deliver the critical health services necessary to achieve SDGs. The situation appears to be especially challenging in many of the low-income countries, as the numbers of workers projected to be supplied as well as the capacity of the countries to employ them will remain well below the WHO SDG threshold of health worker density [13, 31].

However, it should be remembered that the WHO SDG threshold of health worker density is not meant to be taken as an optimal level; there is likely to be considerable scope for improving efficiency of services and productivity of health workers to expand health coverage with fewer numbers of health workers. For example, propagating service delivery models that utilize more lower-skilled cadres (e.g., community health workers, nurses/midwives) may achieve greater coverage for essential primary care services with the same resources used to produce higher skilled, but fewer, physicians and specialists [13]. Many countries are exploring different approaches to health care delivery that will likely lead to fundamental changes in the skills mix and organization of health workers [33]. Increases in productivity might also be achieved through technological advances which could reduce the overall number of health workers demanded and/or shift demand more toward different types of health workers.

In a perfectly fluid market, shifts in demand for health workers would lead to commensurate adjustments in the production (supply) of health workers in order to clear the market. However, neither prices nor the number of workers produced may readily respond to such changes in demand due to rigidities in the health labor market introduced by the influence of labor unions, fee schedules, or professional regulations. While labor unions, professional associations and regulations play a vital role in ensuring the quality and safety of care and protecting the rights of healthcare workers, they may also contribute to unintended delays in responding to changing labor market conditions. This in turn could result in shortages of some categories of workers and oversupply and unemployment among other categories of workers. Addressing these market failures will require appropriate regulation, good governance, and effective collaboration among stakeholders toward the shared goal of improving access to health care for all.

These analyses suggest that in many countries, economic growth alone will not suffice to meet the health worker needs in many of the low-income countries. It cannot be emphasized enough that the health workforce projections presented in this paper assumes *no changes in the technology or efficiency* of the healthcare delivery system: that there will be no changes in the organization of the health care delivery system, or in worker productivity or technology. Thus, these projections do not account for potential changes in productivity due to the engagement of other types and levels of health workers, such as physician assistants, community health workers, and other categories of workers. As more data become available about the numbers and densities of these other types of health workers, future workforce projections can be further refined to include these cadre-specific estimates as well as examine simulations for alternative workforce skills mix models and their implications for service delivery. The supply projections also do not take into account attrition rates in the existing stock of health workers, and the additional number of workers who will need to be educated and employed in order to replace those who exit from the labor market. We also do not consider international migration of health workers, which will affect the distribution of health workers as workers move from low demand to high demand countries.

## Conclusions

The global shortages projected for 2030 may not occur if labor productivity could be increased, for example, through better use of technology, improved skills development, and institutional reforms. A major challenge to the international community is to determine what kind of additional investments will be needed not only to increase the number of health workers in those countries facing health workforce shortages, but also to achieve greater productivity and efficiency with the limited number of available health workers and a more effective distribution and deployment of health workers both within and across countries.

Opportunities exist to bend the trajectory of the number and types of health workers that are available to meet public health goals and the growing demand for health workers. Improvements in health worker productivity supported by technology-driven efficiency gains, changing the skills mix and other cost-savings approaches could potentially lead to fewer health workers needed to provide equivalent levels of health care services. On the other hand, advances in technology could also increase the scope and complexity of healthcare interventions, and may lead to even greater demand for more and higher skilled health workers. With foresight and equipped with an understanding of the future labor market for health workers, more strategic policies can be developed to improve both the supply and distribution of health workers to achieve both public health goals and address economic forces.

## Additional file

Additional file 1: A detailed and technical description of the demand projection methodology, model specification choice, and imputation for missing data is provided in Annex A. Methods detailing the projection of real GDP per capita through 2030 are described in Annex B. Annex C describes the methods used to project private household out-of-pocket expenditures. (DOCX 182 kb)

## Abbreviations

AOWs: All other health workers
DALYs: Disability-adjusted life years
GDP: Gross domestic product
GLM: Generalized linear model
OOP: Out-of-pocket
SDGs: Sustainable Development Goals
UHC: Universal health coverage
USDA: United States Department of Agriculture
WB: World Bank
WHO: World Health Organization

## References

[1] United Nations General Assembly. Transforming Our World: The 2030 Agenda for Sustainable Development [Internet]. United Nations; 2015 [cited 2015 Oct 10]. Available from: http://www.un.org/ga/search/view_doc.asp?symbol=A/70/L.1&Lang=E

[2] Anand S, Bärnighausen T (2012). Health workers at the core of the health system: framework and research issues. Health Policy.

[3] Scheil-Adlung X (2013). Health workforce benchmarks for universal health coverage and sustainable development. Bull World Health Organ.

[4] Campbell J, Dussault G, Buchan J, Pozo-Martin F, Guerra Arias M, Leone C, et al. A universal truth: no health without a workforce [Internet]. Geneva: Global Health Workforce Alliance and World Health Organization; 2013 [cited 2015 Feb 1]. Available from: http://www.who.int/workforcealliance/knowledge/resources/hrhreport2013/en/.

[5] McPake B, Maeda A, Araujo EC, Lemiere C, El Maghraby A, Cometto G (2013). Why do health labour market forces matter?. Bull World Health Organ.

[6] Chen L, Evans T, Anand S, Boufford JI, Brown H, Chowdhury M (2004). Human resources for health: overcoming the crisis. Lancet.

[7] Jimba M, Cometto G, Yamamoto T, Shiao L, Huicho L, Sheikh M. Health workforce: the critical pathway to universal health coverage. Background paper. Montreux, Switzerland; 2010 [cited 2016 Nov 13]. Available from: http://healthsystemsresearch.org/hsr2010/images/stories/10health_workforce.pdf

[8] Kinfu Y, Poz MRD, Mercer H, Evans DB (2009). The health worker shortage in Africa: are enough physicians and nurses being trained?. Bull. World Health Organ.

[9] Scheffler RM, Mahoney CB, Fulton BD, Poz MRD, Preker AS (2009). Estimates of health care professional shortages in Sub-Saharan Africa by 2015. Health Aff (Millwood).

[10] Bruckner TA, Scheffler RM, Shen G, Yoon J, Chisholm D, Morris J (2011). The mental health workforce gap in low- and middle-income countries: a needs-based approach. Bull World Health Organ.

[11] World Health Organization. The World Health Report 2006: Working Together for Health [Internet]. 2006 [cited 2015 Mar 12]. Available from: http://apps.who.int//iris/handle/10665/43432.

[12] Castillo-Laborde C (2011). Human resources for health and burden of disease: an econometric approach. Hum Resour Health.

[13] World Health Organization. Health workforce and services: Draft global strategy on human resources for health: workforce 2030. Rep. Secr. Exec. Board EB13836 [Internet]. 2015 [cited 2015 Dec 20]; Available from: http://apps.who.int/gb/ebwha/pdf_files/EB138/B138_36-en.pdf

[14] McPake B, Scott A, Edoka I. Analyzing Markets for Health Workers: Insights from Labor and Health Economics. Washington: World Bank Publications; 2014.

[15] Araujo EC, Evans TG, Maeda A (2016). Using economic analysis in health workforce policy-making. Oxf Rev Econ Policy.

[16] Scheffler R, Bruckner T, Spetz J (2012). The labour market for human resources for health in low-and middle-income countries.

[17] Andalon M, Fieds G (2013). A different view of the crisis of health care professionals in Africa: a labor market approach.

[18] Vujicic M, Zurn P (2006). The dynamics of the health labour market. Int J Health Plann Manage.

[19] Scheffler RM, Liu JX, Kinfu Y, Poz MRD (2008). Forecasting the global shortage of physicians: an economic- and needs-based approach. Bull World Health Organ.

[20] Hernandez P, Dräger S, Evans DB, Tan-Torres Edejer T, Dal Poz MR. Measuring expenditure for the health workforce: evidence and challenges [Internet]. Geneva: World Health Organization; 2006 [cited 2016 Nov 13]. Available from: http://www.who.int/entity/hrh/documents/measuring_expenditure.pdf?ua=1.

[21] Cooper RA, Getzen TE, Laud P (2003). Economic expansion is a major determinant of physician supply and utilization. Health Serv Res.

[22] Getzen TE, Rossiter L, Scheffler RM (1990). Macro forecasting of national health expenditures. Adv. Health Econ. Health Serv. Res.

[23] Basu K, Gupta A (2004). [A physician demand and supply forecast model for Nova Scotia]. Cah Sociol Demogr Med.

[24] Cooper RA, Getzen TE, McKee HJ, Laud P (2002). Economic and demographic trends signal an impending physician shortage. Health Aff (Millwood).

[25] Newhouse JP (1977). Medical-care expenditure: a cross-national survey. J Hum Resour.

[26] Makridakis S, Wheelwright S, Hyndman RJ. Forecasting: Methods and Applications, 3rd Edition. New York: John Wiley & Sons. Inc; 1998.

[27] United States Department of Agriculture. ERS International Macroeconomic Data Set [Internet]. USDA Econ. Res. Serv. 2014 [cited 2015 Oct 11]. Available from: http://www.ers.usda.gov/data-products/international-macroeconomic-data-set.aspx

[28] United Nations. Probabilistic Population Projections based on the World Population Prospects: The 2012 Revision. [Internet]. Population Division, DESA; [cited 2014 Aug 29]. Available from: http://esa.un.org/unpd/ppp/

[29] World Health Organization. Global Health Observatory of the World Health Organization. [Internet]. n.d. [cited 2014 May 15]. Available from: http://www.who.int/gho/en/.

[30] World Development Indicators [Internet]. 2013 [cited 2015 Oct 11]. Available from: http://data.worldbank.org/data-catalog/world-development-indicators

[31] Scheffler RM, Cometto G, Tulenko K, Bruckner T, Liu JX, Keuffel EL (2016). Health workforce requirements for universal health coverage and the Sustainable Development Goals – Background paper N.1 to the WHO Global Strategy on Human Resources for Health: Workforce 2030.

[32] Buchan J, Wismar M, Glinos IA, Bremner J, et al. Health professional mobility in a changing Europe: new dynamics, mobile individuals and diverse responses. [Internet]. World Health Organization; 2014 [cited 2016 Nov 13]. Available from: http://cdrwww.who.int/entity/workforcealliance/03.pdf.

[33] Lipstein SH, Kellermann AL (2016). Workforce for 21st-century health and health care. JAMA.

