# Erratum to: Global Health Workforce Labor Market Projections for 2030

**DOI:** 10.1186/s12960-017-0193-4

**Published:** 2017-02-20

**Authors:** Jenny X. Liu, Yevgeniy Goryakin, Akiko Maeda, Tim Bruckner, Richard Scheffler

**Affiliations:** 1 Institute for Health and Aging, Department of Social and Behavioral Sciences, University of California, San Francisco, 3333 California Street, Suite 340, San Francisco, CA 94118 United States of America; 2 Health Division, Labour and Social Affairs, Organization for Economic Co-operation and Development, 2 rue Andre Pascal, Cedex 16, 75775 Paris, France; 3 School of Public Health, University of California, 635 E. Peltason Dr., 92697-3957 Irvine, CA United States of America; 4 School of Public Health, Goldman School of Public Policy, University of California, Berkeley, 50 University Hall MC7360, 94704 Berkeley, CA United States of America

## Erratum

After this article [1] was published the authors noticed that the wrong version of Fig. 1 had been uploaded. The Correct figure, is shown below.

**Fig. 1:**
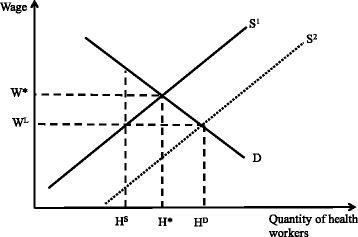
Health worker static labor market theoretical framework. Legend: Demand (D) and supply (S) interact to determine the number of workers (H*) that will be employed at a market wage rate (W*). At a wage rate (W^L^) that is lower than the market optimum (W*), a shortage of workers results, and the number of workers demanded (H^D^) exceeds the number supplied (H^S^). To alleviate shortages in this market, either (1) additional compensation could be given to increase wages to W* and attract more workers into the market, or (2) the production of workers could be increased such that supply shifts outward (S^2^) and the quantity demand (H^D^) is achieved while keeping wages at W^L^
