# Supplementary material for: Global Health Workforce Labor Market Projections for 2030
Source: Hum Resour Health. 2017 Feb 3;15:11. doi: 10.1186/s12960-017-0187-2 (PMC5291995; doi:10.1186/s12960-017-0187-2)
Supplement: Additional file 1: — A detailed and technical description of the demand projection methodology, model specification choice, and imputation for missing data is provided in Annex A. Methods detailing the projection of real GDP per capita through 2030 are described in Annex B. Annex C describes the methods used to project private household out-of-pocket expenditures. (DOCX 182 kb) [file 12960_2017_187_MOESM1_ESM.docx]

**Annex A: Estimation Method for HRH Demand and Supply Projections**

**A1. Methodological overview**

The steps in economic modeling of projected health workforce numbers are described in reference to the illustrative example depicted in Figure A.1. The demand model (D) reflects the number of workers that will be demanded in each country given anticipated economic and demographic conditions. In other words, this is the size of the workforce that a country is likely to be able to afford. We then compare this to the projected supply of health workers based on the historical trend in health workers for each country (S). The surplus or shortage of per capita workers numbers can then be calculated as the difference between what is demanded and what is supplied.

For example in Figure A.1, economic, health spending, and demographic changes may demand only 2.3 health workers per 1000 population by 2030, represented by the scenario D1. Compared to a projected supply of 3.4 physicians per 1000 population in 2030 (S), this would represent a surplus of health workers. In a different scenario, future shortages of health workers could occur in the scenario represented by D2 in which the 2.4 health workers per 1000 demanded in 2015 increases to 4.8 health workers per 1000 in 2030. This translates into a shortage of 0.45 health workers per 1000 in 2015, growing to 1.4 health workers per 1000 in 2030 if nothing is done to actively augment worker supply.

For additional context, Figure A.1 also includes a needs-based (N) estimate that is commonly employed to assess the adequacy of the size of the health care workforce (WHO 2006). The needs-based forecast (N) reflects the number of physicians that would be required to reach a desired benchmark of service utilization (WHO 2006, Scheffler et al. 2013). In our illustrative example, there will be 4.1 health workers per 1000 population that needed to deliver healthcare services at the desired level of coverage, which corresponds to a shortage of health workers based on the need criterion.

We can then multiply this estimated shortage or surplus by projected population numbers to calculate the absolute deficit or excess numbers of health workers.

The steps in estimating the future demand and supply of health workers are as follows:

1. Estimate an economic model that predicts the density of physicians (i.e. per 1000 population) that will be demanded in each country given predicted future growth in income, out-of-pocket (OOP) health expenditures, and size of the population aged 65 or over. A ratio of 2.517 nurses/midwives to physicians is then applied to obtain the numbers of nurses/midwives. Constant multipliers by income level are then applied to the combined demand densities of physicians and nurses/midwives to obtain estimates of all other health worker demand densities. Projected population size is then used to calculate the corresponding numbers of workers that will be demanded. The number of physicians, nurses/midwives, and all other workers (AOWs) are then summed to obtain an aggregate estimate of the number of total health workers demanded.
2. Project the future supply of physicians and nurses/midwives per 1000 population for each country between 2013 and 2030, based on historical trends via a simple linear projection. Constant multipliers by income level are then applied to the combined supply densities of physicians and nurses/midwives to obtain supply density estimates of AOWs. Projected population size is then used to calculate the corresponding numbers of workers of each cadre that could be supplied for each future year.
3. The projected numbers of total health workers demanded in 2030 is then compared to the number supplied in 2030. A country is deemed to have a shortage of health workers in 2030 if the number of workers demanded exceeds the number supplied. The total number of health workers in countries that are predicted to have a shortage in 2030 are then summed by World Bank region and income level.

**Figure A.1 Conceptual framework for projecting health worker labor supply and demand**

It is important to note that demand and supply are endogenously related in the labor market. However, we employ different methods to project each that seeks to address potential endogeneity. Namely, we purposively employ a linear growth projection model for supply that is based solely on an exogenous time trend. Further, all parameter inputs into the demand model are lagged up to five years to ensure the direction of causality.

**A2. Data sources**

The data sources used in this exercise are outlined in **Table A1.** All available data points for all countries/territories and years indicated were downloaded and merged together.

Table A1 Data sources

| **Indicator** | **Years** | **Source** |
| --- | --- | --- |
| Number of physicians and nurse/midwives per 1000 population | 1990-2013 | WHO Global Health Observatory |
| Total population, Population aged 65 or over | 1980-2030 | United Nations, Department of Economic and Social Affairs, Population Division |
| Real GDP per capita (2011 PPP $) | 1995 - 2030 | See Annex 2, estimation by Patrick Eozenou |
| Total health expenditures per capita (PPP constant 2011 international $), Percent of total health expenditures spent out-of-pocket | 1995-2013 | World Development Indicators, extracted from the WHO Global Health Expenditure database |

Data on worker density from the WHO Global Health Observatory database for 193 countries were first cleaned to remove obvious outliers due to misreporting. In each of the country-year observations listed in Table S2, outliers were replaced with missing data so that estimated growth rates would not be unduly influenced by arbitrary substitution.

| **Table A2 Workers per 1000 population data outliers** | |
| --- | --- |
| **Country** | **Years** |
| Bhutan | 2012 |
| Saint Lucia | 1999 |
| Swaziland | 2000 |
| Bahrain | 2005, 2011 |
| Bolivia | 2001 |
| Cabo Verde | 2004 |
| Cameroon | 2004 |
| Central African Republic | 2004 |
| Congo | 1998 |
| Ghana | 2004 |
| Guinea-Bissau | 2004 |
| Guyana | 2004 |
| Micronesia | 2009 |
| Samoa | 1999 |
| Sierra Leone | 2004 |
| Zambia | 2004 |
| India | 1991 |

Missing data points for physicians and nurses/midwives per 1000 population between any two real data points were then linearly interpolated. The resulting dataset of physicians and nurses/midwives per 1000 population that accounts for the data outliers in Table S1 and with interpolated values was used for both worker supply and demand projections.

**A3. Demand model estimation and results**

*Empirical specification*

Previous research has shown that indicators of gross domestic product or national income are the best predictors of health expenditures, of which, labor is the principle component (Cooper et al. 2003, Getzen 1990, Newhouse 1977, Plaff 1990). To our knowledge, few have previously projected future health workforce labor market demand. Owing to data requirements, early works largely focus on specific developed countries for which data on health workers are more readily available (e.g. Korch et al. 2012, Basu & Gupta 2004). Leveraging efforts to obtain cross-national and longitudinal data on health workers, Scheffler et al. (2008) were the first to forecast the demand, need, and supply of physicians for 158 countries with suitable data. While notable in the scope of global coverage, their resulting model relied on only one model parameter input—gross national income—to generate projections.

We build on directly on this previous work. Our demand model projection utilizes per capita indicators of gross domestic product (GDP), based on purchasing power parity, 2011 constant international US dollars, household out-of-pocket (OOP) health expenditures, as well as the size of the population aged 65 or over as the main predictors. We exclude additional structural factors affecting the labor market, such as attrition, training capacity, labor regulations, and migration, as these data are largely unavailable across countries or over time.

Each variable in our demand model is selected based on the following rationale:

- GDP per capita: Overall economic growth is expected to drive demand for health care with a positive elasticity as a normal good. Indicators of economic growth have been found to determine health worker employment (Cooper et al. 2003) and have previously formed the fundamental building blocks of forecast estimates of physician demand by Scheffler et al. (2008).
- Household OOP health spending is included as a proxy measure of the generosity of health insurance coverage within a given country. As such, we expect higher OOP payments to lower the derived demand for health workers per capita; in other words, less generous coverage leaves individuals to pay more out of pocket. While overall healthcare spending may trend upward with national income, the portion spent OOP is largely determined by the level of coverage by health insurance, government subsidies, and other forms of risk pooling and financial protection.
- The size of the population aged 65 or over is included as an indicator of the demographic effects of population aging and ensuing demand for health care services utilized at older ages (Cooper et al. 2002). Driven by lower adult mortality, demographic transitions, particularly of rapidly aging populations in large countries, such as China and India, will place additional pressure on health care services for the elderly.

Using historical data on physician densities, GDP per capita, OOP spending per capita, and the size of the population aged 65 or over, we estimated the relationship between the economic and population indicators and physicians per 1000 population using a generalized linear model (GLM). Missing data points for physicians per 1000 between any two real data points were linearly interpolated. No adjustments were made where data was missing in projected physician densities.

For countries where historical data for total per capita health expenditures and OOP health spending was completely missing, mean yearly values for the specific World Bank region and income group combination to which the country belonged were substituted (see Annex C, below, *Projecting out-of-pocket expenditures*). No adjustments for missing data were made where data was not available for population or GDP per capita; this data tended to be missing for a handful of very small countries, where the projections would minimally affect overall shortage estimates. Thus, countries for which population or GDP per capita data were not available are excluded from the demand projections.

All variables were transformed into logs. To avoid endogeneity, GDP per capita, OOP spending per capita (OOPPC), and the size of the population aged 65 or over (Pop65) were all lagged up to five years to allow time for such factors to work through the economy and affect the labor market, as other authors have done in previous projection exercises (Getzen 1990; Scheffer et la. 2008). A stepwise approach was used to select the specific combination of year lags that maximized the predictive power of each variable. Lagged variables that achieved a minimum 1% level of significance after repeated iteration were kept within the model, resulting in the following optimal model:

(Eq 1) ln(*physicians per 1000 population_it_*) = β_0_ + β_1_*ln(*GDP per capita_it-1_*) + β_2_*ln(*GDP per capita_it-4_*) + β_3_*ln(*GDP per capita_it-5_*) +β_4_*ln(*OOPPC_it-2_*) + β_5_*ln(*Pop65_it-3_*) + *μ_i_* + *ξ_it_*

where *μ_c_* represents a vector of country fixed effects, *ξ_ct_* is the disturbance terms, and β coefficients are unknown parameters to be estimated from the model. Country fixed effects are included to account for time-invariant unobservable heterogeneity across countries (i.e. differences in baseline characteristics between countries). Quadratic terms for income and health spending indicators to additionally account for nonlinearities were investigated but ultimately excluded due to multicollinearity.

*Model specification selection*

Three alternative specifications for the demand model were checked. Specifically, we examined the stability of the results with the following changes:

1. Using the percentage of the population aged 65+ instead of absolute size of population aged 65+.
2. Using OOPPC as a percentage of total per capita health expenditures in place of OOPPC.
3. Using the percentage of the population aged 65+ and OOPPC as a percentage of total per capita health expenditures in place of their respective absolute number counterparts.

To select the appropriate model, the dataset was split into two parts:

1. Initialization dataset included data for years 1995 – 2004.
2. Testing dataset included data for years 2005 – 2013 (except for log physician densities variable, which was set to missing).

Each model specification was first estimated using the initialization dataset, using the lag structure from Equation 1. The estimated parameters were then applied to the actual covariates from the testing dataset to obtain predicted values of physician densities for 2005 through 2013. These predicted values were then compared with actual data for physician densities in 2005-2013. To formally assess the fit of each model, we calculate the mean square root of the squared error:

(Eq 2) Mean error = $\frac{1}{CT}\sum\sqrt{{\hat{{(ln(phys per 1000)}_{ct}}-{ln(phys per 1000}_{ct})}^{2}}$

Table A3 summarizes mean errors resulting from different model specification. The optimal model (Eq 1) was found to generate the best predictions in terms of having the lowest mean errors. Therefore, this model was chosen to estimate parameters using full dataset (1995-2013). Predicted values of logged physician densities from this model were then transformed with an antilog and multiplied by a correction factor ($e^{\sigma^{2}/2}$) to account for the skewed distribution. Future values of physicians per 1000 population were then multiplied by projected total population size (medium fertility assumptions) for each year to obtain the absolute number of physicians.

| **Table A3 Mean errors for demand projection models** | | | |
| --- | --- | --- | --- |
| **Model** | ***N=C*T*** | **Mean** | **Standard deviation** |
| Eq 1 | 653 | 0.2640 | 0.5640 |
| Alternative 1 | 653 | 0.2779 | 0.5403 |
| Alternative 2 | 653 | 0.2649 | 0.5627 |
| Alternative 3 | 653 | 0.2768 | 0.5322 |

The range of demand (Eq. 1) projections at 0.5 S.D. and 1.0 S.D. are shown in Figure A.2, below.

**Figure A.2 Range of Demand Projections within 0.5 and 1.0 Standard Deviation (SD)**


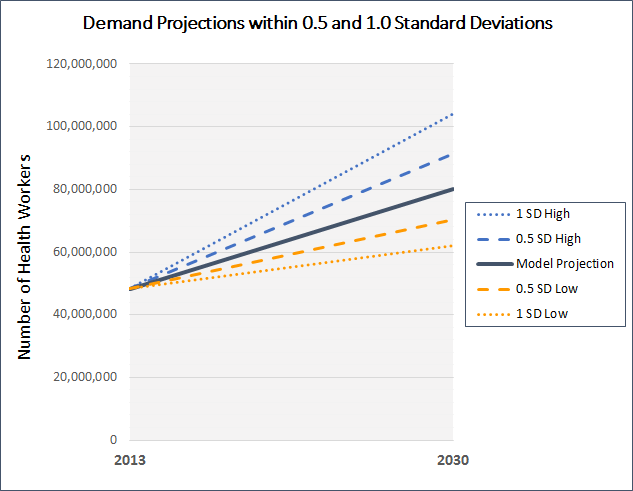


Because the demand model requires rich historical data on health worker densities, separate models for nurses/midwife and all other health professionals could not be estimated; data for these cadres are insufficient to produce demand projections. To obtain the estimates for nurses/midwives, we multiply the projected number of physicians demanded for each country by 2.517, the ratio of nurses/midwives to physicians accepted as a global benchmark (WHO 2016). By using this constant ratio, we assume that the production function for healthcare workers in terms of skills mix stays constant. To obtain estimates of all other workers (AOWs), we apply constant multipliers according to World Bank income level (high income = 0.373; upper middle income = 0.406; lower middle income = 0.549; low income = 0.595) according to the following formula for each country of a given income classification:

(Eq 3) *AOWs per 1000 population* = *multiplier* * [(2.517* *physicians per 1000 population*) + *physicians per 1000 population*]

*Demand model sensitivity analyses*

We conducted two additional sensitivity analyses of the projections of physician demand resulting from the optimal demand model chosen to alternative input parameters.

First, we assess the stability of the predictions to alternative estimated future values of GDP per capita. We use alternative estimated real GDP per capita (US$2010) from the Economic Research Service (ERS) International Macroeconomic Data Set published by the United States Department of Agriculture (USDA), available at <http://www.ers.usda.gov/data-products/international-macroeconomic-data-set.aspx> (Accessed on September 27, 2015). There was a relatively small (9 percent) difference in the total estimated shortages in 2030 based on the two methods (15.6 million with the main method we used, and 17.0 million using USDA numbers)

Second, we examine the possible upper and lower bounds of the predictions older than 65 resulting from high and low future population estimates. Because population is the largest driver of demand in our model, using the high and low variant estimates, we can obtain predicted total health worker deficits that may result from population growth among people older than 65 that is higher and lower than expected, compared to the median estimate that is presented in the main results. These alternative low and high estimates are shown in Figure A1 below, indicating a rather tight band for the resulting predicted values.

**Figure A1. Projected total health worker deficit using low and high projections for the size of the population older than 65**

Note: where the predicted demand was less than the predicted supply, the estimated deficit was assumed to be zero.

**A4. Supply model**

The supply of physicians and nurses/midwives can be projected to 2030 based on historical data on the increase in physician and nurse/midwives densities (i.e. per 1000 population) in each country. Yearly health workforce density data since 1990 are available from the WHO Global Health Observatory database. We extract all available data points for physicians and nurses/midwives.

Various econometric approaches can be used to project supply numbers, each with advantages and disadvantages as described in Table A.5. Of the three types of econometric approaches, th growth rate method is the simplest, most straightforward, and requires the least amount of data, but may be less accurate given stronger functional form assumptions. Other methods rely less on the functional form assumption, but require more data points. A moving average or distributed lag model (2) which gives more weight to more recent data requires that data be available for a continuous number of years, and an ARIMA (autoregressive integrated moving average) model (3) requires that a long time series be available (i.e. ideally back to 1980) with very few missing data points. Given the availability of the health worker data, we chose to proceed with the growth rate model, which has previously been employed in health worker projections (Scheffler et al. 2008).

| Table A.5 Econometric modeling approaches for projecting supply | | |
| --- | --- | --- |
| Model | **Advantages** | **Disadvantages** |
| 1. Growth rate | - Simple and straightforward - Only need 2 data points minimum per country | Potentially less accurate if inappropriate functional form |
| 2. Moving average/ distributed lag | - Gives more weight to recent data - Relies less on functional form assumptions | Requires that the workforce numbers be populated for at least some number of continuous years |
| 3. ARIMA | - Can account for cyclical fluctuations - Relies less on functional form assumptions | Need data for a longer time period (back to 1980) with very few missing observations. |

This growth model assumes that current trends in the growth of physician numbers will continue as they have historically for each country. Using the growth rate approach, we explored two functional forms:

1. Exponential (i.e. log-linear)

(Eq 4a) ln(*Physicians per 1000 population_t_*) = *α*_0_ + *α*_1_**year_t_* + *ε_t_*

(Eq 4b) ln(*Nurses/midwives per 1000 population_t_*I = *β*_0_ + *β*_1_**year_t_* + *ε_t_*

1. Linear

(Eq 5a) ln(*Physicians per 1000 population_t_*) = *α*_0_ + *α*_1_**year_t_* + *ε_t_*

(Eq 5b) ln(*Nurses/midwives per 1000 population_t_*I = *β*_0_ + *β*_1_**year_t_* + *ε_t_*

Equations 4 and 5 were estimated for each country from time *t* = {1990, … 2013} where *ε_t_* is the random disturbance term and *α*_0_, β_0_, α_1_ and β_1_ are unknown parameters, with the last two parameters representing the growth rates to be estimated from the model.

Comparing the resulting projections between these functional forms revealed at the exponential specification yielded estimates that appeared to be magnified (and potentially unrealistic) compared to a linear specification. Coupled with the sparse number of data points for many lower income countries, resulting predicted values appeared to be less stable. Within-sample specification tests were also not possible given the data constraints. We therefore adopted the more conservative linear specification based on a *status quo* scenario which assumes the supply growth is exogenous and only trends with time following the historic trends. This scenario also implies a relatively rigid labor market, which may be plausible for the health labor market that is dominated by strong professional associations.

Equation 5 was estimated for each country, and for physicians and nurses/midwives separately. We then applied the following rules to predict future (2014 – 2030) values of worker densities:

1. Where at least two data points were available, we extended the estimated linear trend into the future, until 2030 using the estimated coefficients for *α* and *β*.
2. If the estimated linear growth was found to be too large or too small, the country’s growth rate was replaced with aggregate medians, and then the median growth rate was applied to the last available observation for that country (i.e. most recent year).
   1. For physicians: If a given country’s linear growth rate was larger or smaller than 1 standard deviation from the mean growth rate for all countries, the region-income group specific median growth rate was substituted.
   2. For nurses/midwives: For nurses, there was large over-dispersion of the linear growth rate distribution. Consequently, if a country’s linear growth rate was larger than 80% or smaller than 20% of the growth rate distribution, then the WB income group specific median growth rate was substituted.[[1]](#footnote-1)
   3. For both physicians and nurse/midwives: If the predicted density in 2030 resulted in a negative number, these country’s growth rate was also replaced with the corresponding median aggregate value.
3. If there was just one point for a country (and thus linear growth rate could not be estimated), we applied the same median substitution for the growth rate as described in 2b.
4. When no observations were available before 2013 (i.e. no empirical data at all for both physicians and nurses/midwives), neither the physician nor nurse/midwives supply was projected. Instead, the mean 2030 predicted supply density across countries of the same income level was substituted.
5. In certain cases, special treatment was given to particular countries because of data problems or predicted growth rates or densities that were implausible.
6. Dominican Republic: The physician growth rate was replaced with the median value.
7. Greece: The nurse/midwife growth rate was applied to the 2000 density value (rather than the 2001 value).
8. Chile: The nurse/midwife growth rate resulted in excessively large predictions; the growth rate was reset to 0.0001/year to reflect a stable trend.
9. Haiti: There was only one historical data point for nurses/midwives. The median income value that would be applied was negative, which resulted in predicted densities near zero; the growth rate was reset to 0.0001/year to reflect a stable trend.
10. Gambia and Kenya: The estimate growth rates were negative, leading to predicted densities near zero; the group median growth rate was substituted.
11. In a number of countries, no empirical data for nurses/midwives were available, but information on physicians was available. We therefore applied to global ratio of 2.517 nurse/midwife-to-physician ratio to obtain the estimate for nurse/midwife density. The countries affected are: Iraq, Slovakia, Bosnia and Herzegovina, Serbia, and Macedonia.

These various substitutions and the number of countries affected are summarized in Table A6.

| **Table A6. Summary of methods used to predicting future worker supply** | | | | | | |
| --- | --- | --- | --- | --- | --- | --- |
|  |  | **Method for addressing missing data** | | | | |
|  | **Total N** | **a** | **b** | **d** | **e** | **f** |
| **Physicians** |  |  |  |  |  |  |
| Supply | 208* | 136 | 50 | 21 | 1 | 0 |
| Demand | 165 | 120 | 44 | 0 | 1 | 0 |
| **Nurses/midwives** |  |  |  |  |  |  |
| Supply | 208* | 81 | 100 | 19 | 3 | 5 |
| Demand | 165 | 73 | 84 | 0 | 3 | 5 |
| * Note that although 201 countries are included in the analysis, 2030 supply predictions are only made for 208 countries. Kosovo and St. Martin (French part) are excluded from the projections because they do not have population estimates for 2030. | | | | | | |

The projected supply of physicians and nurses/midwives per 1000 population for each future year was then multiplied by projected population (medium fertility assumptions) in that year to obtain the absolute numbers of physicians and nurses/midwives. The formula is as follows:

(Eq 6) *Number of workers* = *worker per 1000 population* * *population in 2030*/1000

To obtain estimates for the other cadres, we apply the standardized approach adopted by the team and which was previously applied to the need-based worker estimates. The income group-specific multiplier[[2]](#footnote-2) was multiplied with a constant ratio of 3.517 and the physician density, according to the following formulas according to the method applied by WHO (2016):

| High income | Other cadres per 1000 = 0.373 * (3.517) * physicians per 1000 |
| --- | --- |
| Upper middle income | Other cadres per 1000 = 0.406 * (3.517) * physicians per 1000 |
| Lower middle income | Other cadres per 1000 = 0.549 * (3.517) * physicians per 1000 |
| Low income | Other cadres per 1000 = 0.595 * (3.517) * physicians per 1000 |

The resulting density of other cadres was then multiplied by population size (see Eq 15) to obtain the absolute number of workers in other cadres.

**A5. Calculating worker surplus or shortages**

The surplus or shortage of workers for each country *c* and year *t* is calculated as the difference between what is demanded what is supplied:

(Eq 7) *Difference (Surplus/Shortage)_ct_* = *Workers supplied_ct_* – *Workers demanded_ct_*

**A6. Aggregation by region and income level**

We report projected health worker demand, supply, and shortage estimates by regions and income levels as defined by the World Bank (see data.worldbank.org/about/country-and-lending-groups).

Demand projections were only made for 165 countries for which sufficient data for input parameters were available. In reporting aggregate shortages, we only include countries for which there is a projected demand-based shortage in 2013 or in 2030. In other words, countries that are estimated to have health worker surpluses in 2013 or in 2030 are not counted toward shortage totals in respective years (i.e. a zero shortage is assumed). This is consistent with the assumption that there are no transnational movements of workers (i.e. shortages and surpluses do not net out across countries), following the approach of WHO (2006).

**Annex B: Estimation of GDP Projections to 2030[[3]](#footnote-3)**

Estimation Method

- Data from 1995 to 2020 are obtained from the World Economic Outlook database.
- Data from 2014 to 2020 are IMF estimates.
- Data from 2020 to 2035 are projections based on the following assumptions:

1. Cobb-Douglas production function with constant returns to scale: Y = A*K^(1-alpha)*L^(alpha)
2. Labor share (alpha) = 1/3
3. TFP growth (A) and Capital growth (K) are constant at steady state
4. Labor force growth is equal to population growth
5. Data on population growth are taken from the UN-DESA World Population Prospects 2012 (medium fertility assumption)

Steps

1. Derive the projections for annual growth rates of real GDP --> (World Economic Outlook data base (WEO_NGDP_RPCH)
2. Rebase the WDI constant GDP series (2011 GDP deflator = 100) in LCU --> WDI_GDP_constant_LCU2011
3. Derive the projections for real GDP at 2011 prices using projected growth rates (2021-2035) to real GDP estimates from 2020 onward --> REAL GDP PER CAPITA
4. Apply WPP 2012 population projections based on medium fertility assumption to derive projected real GDP per capita (expressed in million LCU per capita) --> REAL GDP PER CAPITA
5. 2011 PPP factors are applied to convert constant GDP in LCU to international $ --> REAL GDP PER CAPITA
6. A multilevel model with country and region random effects is estimated to derive GNI projections from GDP projections --> REAL GNI PER CAPITA

**Annex C: Projecting out-of-pocket expenditures**

Future values of OOP spending per capita are needed as input parameters into the demand model projections to predict future values of physicians per 1000 population. Based on historical data for total health expenditures per capita (PPP constant 2011 international $) and the percentage of total health expenditures spent OOP, we calculate the OOP health expenditures per capita for each country from 1995 to 2013 based on the following formula for each country *c* in year *t*:

(Eq 8) *OOPPC_ct_* = *% total health expenditures spent OOP_ct_***Total health expenditure per capita_ct_*

For 27 countries where health expenditure information was completely missing, year-specific mean OOP spending per capita by region and income group were substituted. These countries were as follows: American Samoa, Aruba, Bermuda, Cayman Islands, Channel Islands, Curacao, Faeroe Islands, French Polynesia, Greenland, Guam, Hong Kong, Isle of Man, DPRK, Kosovo, Liechtenstein, Macao, New Caledonia, Northern Mariana, Puerto Rico, Sint Maarten, Somalia, St. Martin, Taiwan, Turks and Caicos, Virgin Islands, West Bank and Gaza, and Zimbabwe.

To select the appropriate model, the data was split into two parts:

1. Initialization dataset included data for years 1995 – 2008.
2. Testing dataset included data for years 2009 – 2013 (except for OOPPC variable, which was set to missing).

To project future values of OOP spending per capita, we tested eight models with the initialization dataset:

1. For each country *c*, a moving average for 18 prior periods without weights was estimated:

(Eq 9) ${OOPPC}_{ct}=\frac{1}{18}\sum_{x=1}^{18} {OOPPC}_{c,t-x}$

A total of 18 previous periods was chosen to maximize the number of prior year observations possible to base projections off of (e.g. the projection for 2030 needs at least 17 lags to use prior information from 2013).

1. For each country *c*, a moving average for 14 prior periods with weights was estimated, with the more distant observations progressively receiving smaller weights:

(Eq 10) ${OOPPC}_{ct}=\frac{1}{171}\sum_{x=1}^{18} {(18-x)OOPPC}_{c,t-x}$

1. For each country *c*, double-exponential smoothing over 14 prior periods was applied:

(Eq 11) *S_ct_*^[2]^ = α*S_ct_* + (1-α)*S_c,t-1_*^[2]^,

where *S_ct_*^[2]^ is the smoothed original series from *S_ct_* = α*X_ct_* + (1-α)*S_c,t-1_*, and α is the smoothing parameter estimated by minimizes the in-sample sum-of-squared predicted errors; *X_ct_* is the original series.

1. For each country *c*, we estimated the following regression where β represents the yearly difference in OOPPC over the previous year, and ε*_ct_* is a random error term:

(Eq 12) *OOPPC_ct_* = α + β*Year trend_ct_* + ε*_ct_*

1. For each country *c*, we estimated the following regression that additionally accounts for non-linearities in the time trend of OOP:

(Eq 13) *OOPPC_ct_* = α + β_1_*Year trend_ct_* + β_2_(*Year trend_ct_)*^2^ + ε*_ct_*

1. For all countries *c*, we estimated the following pooled regression that includes country fixed effects (γ_c_) as well as a flexible time trend:

(Eq 14) *OOPPC_ct_* = α_c_ + β_1_*Year trend_ct_* + β_2_(*Year trend_ct_*)^2^ + γ_c_ + ε*_ct_*

1. For all countries *c*, we estimated the following pooled regression that includes three dummy indicators for World Bank (W_r_) income group classifications as well as a flexible time trend:

(Eq 15) OOPPC_crt_ = α_r_ + β_1_*Year trend_crt_* + β_2_(*Year trend_crt_*)^2^ + δ_r_ + ε*_ct_*

1. The same regression for Equation 9, but correcting for AR1 serial correlation.

The estimated parameters were then applied to the testing dataset to obtain predicted values for 2009 through 2013. These predicted values were then compared with actual data in 2009-2013. To assess the fit of each model, we again calculate the mean square root of the squared error, displayed in Table A4.

(Eq 16) Mean error = $\frac{1}{CT}\sum\sqrt{{\hat{{(OOPPC}_{ct}}-{OOPPC}_{ct})}^{2}}$

| **Table A4 Mean errors for out-of-pocket health spending projection models** | | | |
| --- | --- | --- | --- |
| **Model** | ***N=C*T*** | **Mean** | **Standard deviation** |
| 1 | 1075 | 104.7602 | 120.2658 |
| 2 | 1075 | 87.52218 | 101.3041 |
| 3 | 1070 | 41.89035 | 77.43136 |
| 4 | 1075 | 218.3716 | 173.5767 |
| 5 | 1075 | 225.382 | 166.4547 |
| 6 | 1075 | 100.8074 | 81.81804 |
| 7 | 1040 | 147.0227 | 134.1653 |
| 8 | 1075 | 94.51554 | 76.15938 |
| 9 | 1075 | 44.60224 | 58.05829 |

Models 2 and 3 (Table A4) were found to have the smallest prediction error among the first eight. Moreover, we average the predictions resulting from Methods 2 and 3, which can improve prediction accuracy (Makridakis et al., 2008). To further improve prediction accuracy, we have selected the optimal method (i.e. which minimizes the square root of the squared error) for each country, rather than across all observations. Thus, for example, method 9 may minimize prediction errors in country A, but weighted average may minimize error in country B. Rather than force one method on all countries uniformly, we have allowed the optimal model selection to be country-specific. This indeed has led to further substantial reduction in the average prediction error across all observations (with mean squared error now being 22.0), and therefore this is our preferred approach.

The predicted values for *OOPPC* were then used as inputs into the overall demand model. In six countries (Brunei, Burundi, Cote d'Ivoire, Lebanon, Qatar, Serbia), the OOPPC projection using the combined method yield predicted values below zero for certain future years. For these countries, estimates from Model 2 alone were used as the projected values.

1. Note, because of empirical data for nurses/midwives has more missings, growth rates could be estimated from the historical trend for comparatively fewer numbers of countries. A higher level of aggregation is needed (i.e. income level, rather than region and income level) to obtain suitable median substitution values across a sufficient number of countries within the grouping. [↑](#footnote-ref-1)
2. The multiplier was determined via a separate methodology within the needs-based worker threshold analysis (WHO 2016). [↑](#footnote-ref-2)
3. GDP estimation for 2030 was prepared by Patrick Eozenou (Health Economist, World Bank) for the working paper by Olusoji Adeyi, Caroline Ly, Patrick Eozenou, Allyala Nandakumar, Ariel Pablos-Mendez and Timothy Evans, "The economic transition of health in Africa: a call for progressive pragmatism to shape the future of health financing". [↑](#footnote-ref-3)
